# High Electromechanical Coupling Coefficient of Longitudinally Excited Shear Wave Resonator Based on Optimized Bragg Structure

**DOI:** 10.3390/mi14112086

**Published:** 2023-11-11

**Authors:** Zhiheng Zhang, Weipeng Xuan, Hong Jiang, Weilun Xie, Zhaoling Li, Shurong Dong, Hao Jin, Jikui Luo

**Affiliations:** 1 Ministry of Education Key Laboratory of RF Circuits and Systems, College of Electronics & Information, Hangzhou Dianzi University, Hangzhou 310005, China; 2 Key Laboratory of Advanced Micro/Nano Electronic Devices & Smart Systems of Zhejiang, College of Information Science & Electronic Engineering, Zhejiang University, Hangzhou 310027, China; dongshurong@zju.edu.cn (S.D.); hjin@zju.edu.cn (H.J.)

**Keywords:** single-crystalline lithium tantalate thin film, SM-YBAR, optimized Bragg structure, high electromechanical coupling

## Abstract

In this work, a longitudinally excited shear-wave resonator (YBAR) based on single-crystalline lithium tantalate (LiTaO_3_, LT) thin film is proposed. The YBAR has a 200 nm X-cut thin film and molybdenum electrode. A high effective electromechanical coupling coefficient (k^2^_eff_) of up to 19% for the suspension-type structure was obtained. Furthermore, a Bragg reflector (SiO_2_/Pt) with optimized layer thickness ratio was employed to improve the performance of the YBAR. Compared to the acoustic wave resonators with the conventional quarter-wave (λ/4) Bragg reflector, the proposed YBAR with an optimized Bragg reflector can reflect both the longitudinal and shear waves efficiently, and resonators with spurious-free response and high quality (Q) value were achieved. This work provides a potential solution to enabling high coupling micro-acoustic resonators with high Q factor in the 5G/6G communication system.

## 1. Introduction

Micro-acoustic devices based on the piezoelectric principle (e.g., duplexers, oscillators, transducers, resonators, filters) have been widely used in the communication industry. Currently, filters used in cellular communication are mainly based on the SAW (surface acoustic wave) or BAW (bulk acoustic wave) resonators [1]. The primary challenge for the SAW and BAW filters is the persistent demands for improved performance (e.g., wideband, compact size, lower insertion loss and cost-effectiveness) at higher operating frequency. Achieving these objectives is difficult, as they often conflict with one another. For instance, reducing the size of filters is essential for modern portable terminals, but this can lead to increased insertion loss. Thus, trade-offs between these objectives must be made for the design of high-performance filters. Additionally, the demand for SAW and BAW filters in emerging applications like the Internet of Things (IoT) and autonomous vehicles brings new challenges. These scenarios require filters with unique specifications and capabilities, necessitating innovation and adaptation in filter design. As the fundamental frequency for communication is increasing steadily, traditional SAW filters are gradually being replaced with BAW filters due to their frequency limitations and relatively lower power capacities. 

For micro-acoustic filters, compact size, high operating frequency, large bandwidth, low temperature drift, small insertion loss, and high-power capacity are the requests for the development. BAW filters based on aluminum nitride (AlN) have been commercialized for decades. However, the small electromechanical coupling coefficients (k^2^_eff_) of AlN piezoelectric film and the stringent frequency specifications in some bands for applications (e.g., N77 and N79) are beyond the capability of conventional BAWs, hampering the applications of AlN-based devices in wideband scenarios. Some promising solutions have been explored such as using Sc doped AlN (AlScN) [2,3], or single-crystalline lithium niobate (LiNbO_3_, LN) and lithium tantalate (LiTaO_3_, LT) thin films to enhance the electromechanical coupling coefficient, k^2^_eff_. The introduction of longitudinally excited shear-wave resonators (XBAR or YBAR) represents an attractive development in the field of acoustic wave filters. In recent years, acoustic wave resonators based on single-crystalline LiNbO_3_ and LiTaO_3_ thin films have been investigated by many researchers, and found to be able to meet the requirements for larger coupling coefficients at higher operating frequencies. These resonators typically consist of interdigital transducers (IDTs) on thin piezoelectric plates. Their low loss and decent piezo-coupling characteristics are good for wideband 5G cellular and next-generation Wi-Fi filters. Koulakis and coworkers reported a filter based on the XBARs, which present operating frequencies >5 GHz and have ultra-large bandwidths of ~1.2 GHz [4]. Yandrapalli and coworkers manufactured XBAR filters with a large bandwidth of above 10% at 4.8 GHz and a low insertion loss of ~1.4 dB [5]. Plessky et al. investigated a YBAR based on a Y-cut LiNbO_3_ thin film, which has an ultrawide resonance and anti-resonance distance (up to 22%), corresponding to an extraordinarily high k^2^_eff_ of 60%. Qin et al. proposed a X-cut LiNbO_3_ based YBAR with a solid mounted (SM) structure, which has an effective electromechanical coupling coefficient of up to 50% [6]. Meanwhile, Kadota et al. reported a number of LiNbO_3_ and LiTaO_3_ thin films-based resonators with high performances [7,8,9,10,11]. The reported studies have demonstrated the potential of single-crystalline LiNbO_3_ and LiTaO_3_ thin films for high frequency and wideband applications for next-generation wireless communication. Currently, two primary configurations have dominated in the filter design: the cavity-type and the Bragg reflector-type. Both can constrain acoustic waves within the piezoelectric films. The cavity resonators are renowned for their high-quality factor, which translates to ultra-small insertion loss within the passband and superior frequency selectivity. These features are especially important for applications in that ultra-low power consumption is required. However, since the employment of suspended thin films, the cavity-type resonators are mechanically fragile and susceptible to perturbations, making them less reliable. The Bragg reflector-type resonators are featured with their superior mechanical robustness. Typically they consist of a reflector with alternating high and low acoustic impedance layers, which can create bandgaps for specific acoustic waves, allowing for the selective transmission or reflection of waves with specific frequencies [12,13]. However, the traditional λ/4 Bragg reflector can only reflect one type of acoustic wave well (λ is the wavelength of the acoustic wave in each layer), leading to the leakage of acoustic waves with different modes and the generation of a spurious response. Therefore, it is necessary to develop new types of Bragg reflectors for resonators for the forth-coming communications.

**Figure 1:**
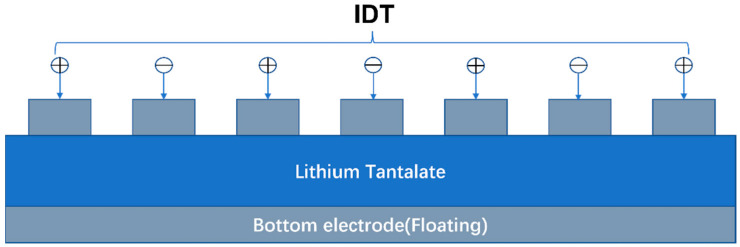
Structure of the suspended YBAR, composing of a piezoelectric layer, an IDT electrode on the surface and a floating bottom electrode.

In this paper, we proposed a YBAR based on X-cut LiTaO_3_ thin film with high electromechanical coupling coefficient. The structure of the YBAR is shown in Figure 1. Similar to a SAW device, it has interdigital transducer electrode formed on surface of the piezoelectric substrate, but it has a floating electrode at the bottom of the thin piezoelectric plate. We also studied several YBARs with optimized Bragg reflectors. Compared to resonators with a traditional λ/4 Bragg reflector, the newly developed YBAR can efficiently reflect both the longitudinal and shear waves. In addition, the proposed resonators possess a higher Q factor and a smoother spectrum with spurious-free characteristics over a wide frequency range.

The general structure of this paper is as follows: in the first section, the effect of different crystal tangents of lithium tantalate on the k^2^_eff_ was investigated. Then, we analyzed the effect of electrode width on the resonant frequency and the k^2^_eff_ for the YBAR structures. The third section dealt with the influence of piezoelectric layer thickness on resonant frequency with different electrode configurations. Section IV delved into the fundamental principles for optimizing Bragg reflectors, at the foundation of the optimization and analysis of the Bragg reflector thickness; the impedance curves and Q values of the conventional and optimized structures were compared. In the fifth part, the simulation results of the optimized Bragg structure and the conventional structure are analyzed in the 3D environment. In this work, the material parameters used for simulation are shown in Table 1.

## 2. Effect of Propagation Direction on Electromechanical Coupling Coefficient

**Figure 2:**
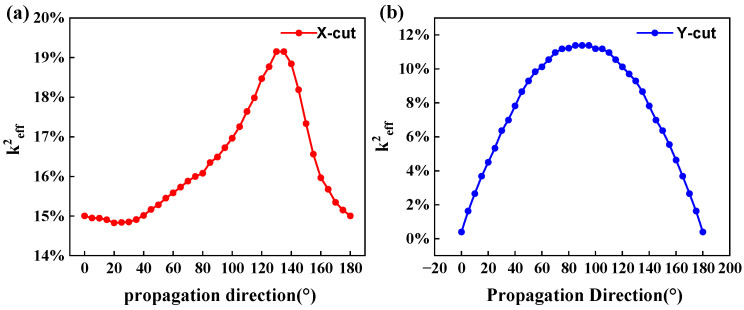
Influence of propagation angle on electromechanical coupling coefficients: X-cut (**a**) and Y-cut (**b**) lithium tantalate thin films.

Electromechanical coupling coefficient, k^2^_eff_, of resonators are related to several aspects. One critical factor that dictates the electromechanical coupling coefficient is the crystal orientation, as piezoelectric properties of the materials are strongly dependent on crystal orientation. The k^2^_eff_ values were calculated and analyzed for both the X-cut and Y-cut cases with different propagation angles. COMSOL Multiphysics was used to conduct the finite element analysis. In the simulation, the thickness of the LiTaO_3_ layer was fixed at 200 nm, and the thickness and width of the molybdenum (Mo) electrodes were 50 nm and 500 nm, respectively. The metallization ratio of the devices was fixed at 50%, yielding a wavelength of *λ* = 2 μm. To save computation resources, the periodic boundary conditions were applied to the four surfaces normal to the main plane. The variations in k^2^_eff_ are shown in Figure 2. For the X-cut LiTaO_3_, the electromechanical coupling coefficient shows a peak around at the 130° propagation angle with a value of 19%; For the Y-cut LiTaO_3_, the maximum k^2^_eff_ can be obtained near the 90° propagation angle with a value of 11%. For the Z-cut LiTaO_3_ (not shown), it is almost zero. Therefore, in the subsequent work, in order to obtain high effective electromechanical coupling coefficients, LiTaO_3_ thin film with X-cut and 130° propagation angle was chosen for the simulation.

## 3. Effect of Electrode Width on Resonant Frequency and k^2^_eff_

**Figure 3:**
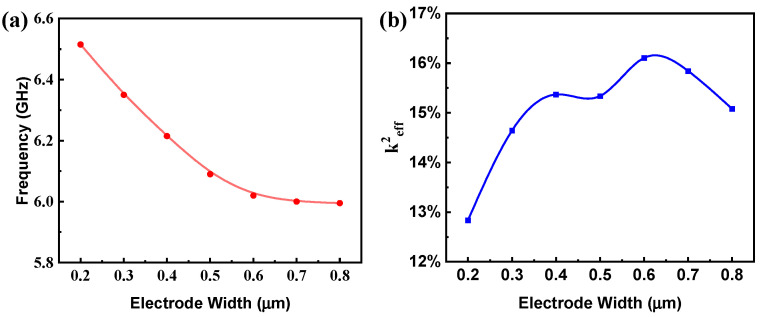
Effects of electrode width on resonant frequency and electromechanical coupling coefficient of a YBAR. (**a**) frequency, (**b**) k^2^_eff_.

This subsection focuses on the effect of electrode width on the resonant frequency and electromechanical coupling coefficient for the YBAR structure. The wavelength of the resonator was fixed at 2 μm and the electrode width varied in the range from 0.2 μm to 0.8 μm. Figure 3a,b present the effect of electrode width on resonant frequency and k^2^_eff_ for the YBARs, respectively. With a narrow electrode width of 0.2 μm, the resonant frequency exceeds 6.5 GHz; when it increases to 0.8 μm, the resonant frequency is approximately 6 GHz. The resonant frequency gradually decreases with the increase in electrode width. The k^2^_eff_, on the other hand, roughly increases with the electrode width when it is smaller than 0.6 μm and then decreases with increasing electrode width.

## 4. Analysis of Resonant Frequencies for Different LiTaO_3_ (LT) Thicknesses

**Figure 4:**
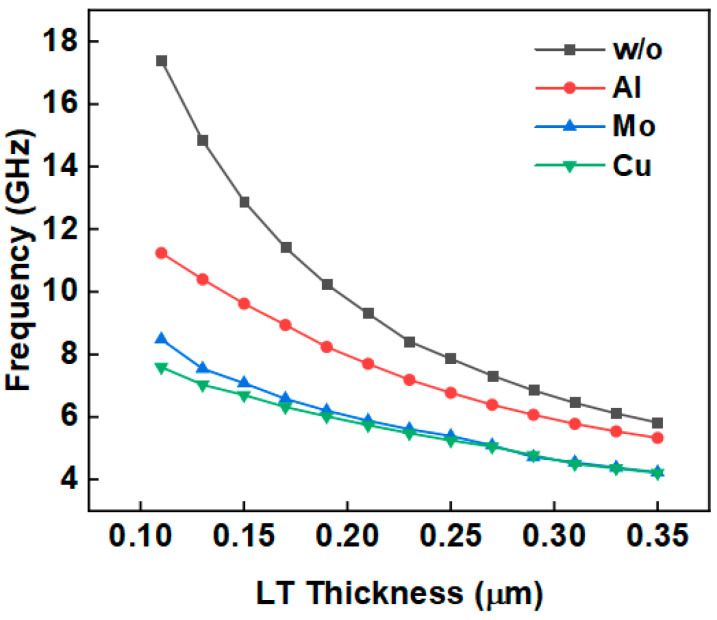
Effect of LT thickness on resonant frequency.

The electrode thickness was set to 50 nm and the width to 500 nm. The cases with Al (aluminum), Mo, Cu (copper) electrodes and no electrode were evaluated. The thickness of the piezoelectric layer was changed from 110 nm to 350 nm. Figure 4 shows that the resonant frequency decreases with the increase in piezoelectric layer thickness. As the density of Al is smaller, the Al electrode is lighter than those of Mo and Cu electrodes with the same volume; the YBARs based on Al IDTs present higher resonant frequencies than those with Mo or Cu electrodes with the same piezoelectric layer thickness due to the mass loading effect. When the thickness of LiTaO_3_ is 200 nm, the resonant frequencies of Mo- and Cu-based YBARs are 6 GHz, while the Al-based YBAR resonates above 8 GHz.

## 5. Bragg Reflector Design and Optimization

Suppressing spurious modes to maintain relatively clean passbands has always been a challenge for the development of acoustic wave resonators, and it is difficult to achieve a spurious-free response for ultra-wideband scenarios. Employing a Bragg reflector is beneficial for achieving a high Q factor, and spurious modes can be suppressed to some extent. 

The solidly mounted resonators (SMRs) are indispensable components in various electronic devices and systems, such as filters, oscillators, and sensors, where enhancing their quality factor plays a pivotal role in achieving superior performance. However, traditional SMRs face inherent limitations stemming from substrate losses, which are primarily attributed to the classical λ/4 reflector stacks employed. For traditional SMRs, their quality factors often fall short of the desired values due to the inherent drawbacks of the reflector stacks. Conventionally, SMRs utilize a quarter-wave reflector stack targeted at reflecting the predominantly longitudinal acoustic waves, which are the dominant mode for many resonators. Unfortunately, this stack design fails to address the comprehensive range of acoustic wave modes, particularly the shear waves. The shear waves exhibit entirely different propagation characteristics compared to longitudinal waves and cannot be efficiently reflected by the standard quarter-wave reflector stack. As a result, these residual shear waves contribute to energy losses of acoustic resonators, which inevitably diminishes the quality factor. Even worse, they generate spurious responses over a wide frequency range. This shortage has stimulated researchers and engineers to explore innovative solutions to optimizing the reflective stack design.

Conventional structures use a quarter-wavelength reflector stack, where the reflective stack is composed of alternating layers with low and high acoustic impedances. With a high acoustic impedance ratio (high acoustic impedance value/low acoustic impedance value), a large reflection coefficient can be obtained, which is beneficial for minimizing acoustic wave leakage into the substrate, thus achieving a high Q factor. For the optimal reflector, the layer thickness *t* is generally one-fourth of the wavelength *λ* at the resonant frequency $f_{R}$:

(1) $$t=\frac{\lambda }{4}$$

The *λ*/4 structure can only reflect a specific wave (i.e., the longitudinal wave), while the transverse waves will still leak into the substrate, leading to extra loss. In order to obtain high-Q devices, the reflective stack should enable the reflection of both wave modes effectively.

Herein, we adopted a design that can efficiently reflect both the longitudinal and transverse waves; the design principle is detailed as follows [15].

According to the blocking band theory of optical films, in a multilayer Bragg mirror, when the phase of each layer drops to the so-called base point (i.e., the transmission minimum), the reflective layer structure also generates a maximum reflection at

(2) $$\phi_{n}=\frac{n\pi }{1+c}$$

where n is an integer and *n* ≤ *c*, $c=\phi_{H}/\phi_{L}$, and the $\phi_{H}$ and $\phi_{L}$ are the acoustic wave phase shifts of the high- and low-impedance layers, respectively. Strictly speaking, the value of *c* should be an integer; however, it can be a non-integer at the cost of a change in reflection coefficient. Still, from the process point of view, selecting a larger *c* will result in a relatively thicker layer, leading to a higher processing cost, which is undesirable, so *c* and *n* should be kept to a minimum value.

In addition, $\phi_{H}$ and $\phi_{L}$ should satisfy the maximum reflection condition with

(3) $$\phi_{H}+\phi_{L}=(1+c)\phi_{n}=n\pi .$$

For the high- and low-impedance layers, the following conditions should be satisfied.

(4) $$\phi_{L-longwave}=\frac{n\pi }{1+c}$$

(5) $$\phi_{H-longwave}=\frac{nc\pi }{1+c}$$

Setting this frequency equal to a desired frequency $f_{R}$ at which an ideal reflection is desired, we can calculate the thickness of the *H*-layer and *L*-layer as:

(6) $$t_{L}=\frac{\phi_{L-longwave}}{2\pi }\times \frac{v_{L-long}}{f_{R}}=\frac{n}{2(1+c)}\lambda_{L-long}$$

(7) $$t_{H}=\frac{\phi_{H-longwave}}{2\pi }\times \frac{v_{H-long}}{f_{R}}=\frac{nc}{2(1+c)}\lambda_{H-long}.$$

where $v_{L-long}$, $v_{H-long}$ are the longitudinal velocities in the low-impedance and high-impedance layers, respectively, and the $\lambda_{L-long}$, $\lambda_{H-long}$ correspond to the wavelengths. Meanwhile, the phase shifts of the transverse wave corresponding to the low- and high-impedance layers are:

(8) $$\phi_{L-shearwave}=\frac{n\pi }{1+c}(\frac{v_{L-longwave}}{v_{L-shearwave}})=\frac{n\pi }{1+c}\times K_{L}$$

(9) $$\phi_{H-shearwave}=\frac{n\pi }{1+c}(\frac{v_{H-longwave}}{v_{H-shearwave}})=\frac{n\pi }{1+c}\times K_{H}$$

where $v_{L-shearwave}$, $v_{H-shearwave}$ are the transverse velocities in the low- and high-impedance layers, respectively. The $K_{L}=\frac{v_{L-longwave}}{v_{L-shearwave}}$ and $K_{H}=\frac{v_{H-longwave}}{v_{H-shearwave}}$ are the ratios of the longitudinal to transverse waves of the reflective stacks.

The optimized reflection can be obtained when both the $K_{L}$ and $K_{H}$ values are equal to two. In reality, the ratios are not strictly equal to two, and the deviation can be evaluated based on a detuning parameter (Δφ). In the following design, the detuning parameter is introduced to optimize the thickness in order to efficiently reflect both the longitudinal and transverse waves. Assuming that there is phase deviation Δφ for both the high and low impedance layers, Equation (3) can be rewritten as:

(10) $$\phi_{L-longwave}+\phi_{H-longwave}=\pi +\Delta \varphi$$

(11) $$\phi_{L-shearwave}+\phi_{H-shearwave}=2\pi -\Delta \varphi$$

(12) $$\phi_{L-longwave}(1+K_{L})+\phi_{H-longwave}(1+K_{H})=3\pi$$

(13) $$\phi_{L-longwave}(1+c+K_{L}+K_{H})=3\pi$$

Therefore, the thickness of the low- and the high-impedance layers can be calculated by:

(14) $$t_{L}=\frac{3/2}{1+c+K_{L}+cK_{H}}\lambda_{L-longwave}$$

(15) $$t_{H}=\frac{3c/2}{1+c+K_{L}+cK_{H}}\lambda_{H-longwave}$$

**Figure 5:**
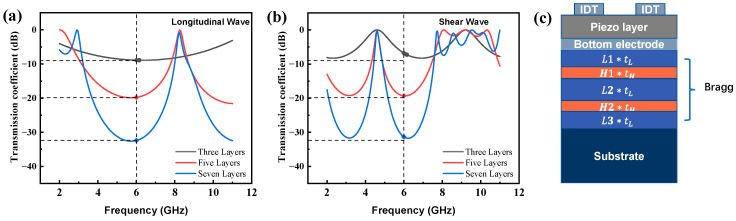
SM-YBAR structure diagram. The transmission coefficient of the longitudinal wave (**a**) and shear wave (**b**), and the Bragg structure (**c**).

In order to reflect the downward propagating waves with a high proportion, a large acoustic impedance ratio between the high and low impedances is required. In this work, the designing working frequency was 6 GHz, so a Bragg reflective layer that works at 6 GHz was designed. The electrode width and the gap were fixed equally at 500 nm. Mo electrodes were chosen for both the upper and lower electrodes, with a thickness of 50 nm and a finger width of 500 nm, and the piezoelectric material was X-130°Y lithium tantalate with a thickness of 200 nm. For the Bragg reflective layer, the transmission coefficients of three, five and seven layers of the longitudinal and shear waves are shown in Figure 5a,b, respectively. According to the results, the reflector consisting of five layers of SiO_2_ (low-impedance) and Pt (high-impedance) can achieve a high reflectivity. For the substrate, monocrystalline silicon with a 5 μm thickness was used, and the thickness of the bottom perfect matching layer was set to be 2 μm. Loss conditions, including the mechanical damping, the dielectric losses, and other losses, were not considered in the simulation. In order to minimize the deviation, the algebraic average ratio of *K_L_* and *K_H_* was used in Equations (14) and (15). In the study cases, the optimized *t_L_* and *t_H_* were set to be 179 nm and 183 nm, respectively. Meanwhile, the L2H thickness ratio configuration was used, corresponding to the thickness coefficients in the following sequence: L1 = L3 = 1, H1 = H2 = 0.5, L2 = 2.

Figure 5c illustrates the configuration of the SM-YBAR, where $t_{L}$ and $t_{H}$ are the calculated layer thicknesses, while the $L\left(i\right)\;and\;H(i)$ are the thickness coefficients (i=1,2…) for each layer.

**Figure 6:**
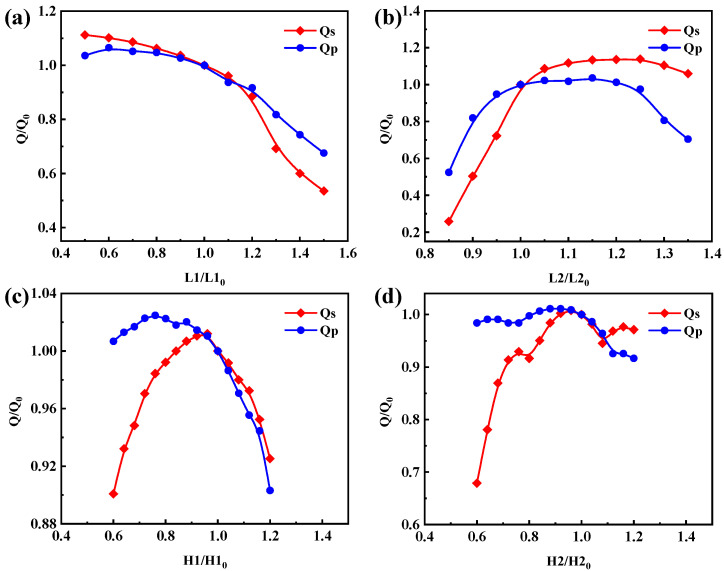
Effect of variation in thickness coefficient of different layers on the Qs and Qp. (**a**) Layer L1, (**b**) layer L2, (**c**) layer H1, (**d**) layer H2.

Figure 6 represents the effect of thickness variation of different layers on Q value, where the horizontal coordinates represent the thickness coefficients, i.e., L1, L2, H1, and H2 (L3 has a negligible effect on the Q value, so it is not plotted). The *y*-axis corresponds to the ratio of the thickness coefficient change in the Q value and the initial thickness coefficient device Q value to the initial thickness coefficient device Q_0_. Q_0_ is the calculated Q value in the original L2H configuration. In the L2H structure configuration, Qs (quality factor at $f_{s}$) and Q_p_ (quality factor at $f_{p}$) are 1502 and 2876, respectively. As shown in Figure 6a, when the thickness factor L1 increases by 20%, Qs and Qp decrease to 0.885 and 0.917 of the original Q_0_. As shown in Figure 6b, as L2 increases by 20%, Qs and Qp increase to 1.136 and 1.012 of the original Q_0_. The influences of H1 and H2 are presented in Figure 6c,d. It can be seen that the thickness variation of each layer has a significant influence on the Q factor of the devices, which provides a reference for the subsequent optimization. The influence of parasitic capacitances introduced by the Bragg reflectors should also be evaluated. For example, considering the second low impedance layer (L2*t_L_), since the upper and lower high impedance layers are metal, and capacitance is introduced in combination with this layer, the thickness of this dielectric layer will affect capacitance, and extra resonance can be generated, leading to the shifting of the optimum reflection point, and finally impacting the Q value of the resonator. The parasitic capacitance needs to be made as small as possible to eliminate this undesirable effect.

**Figure 7:**
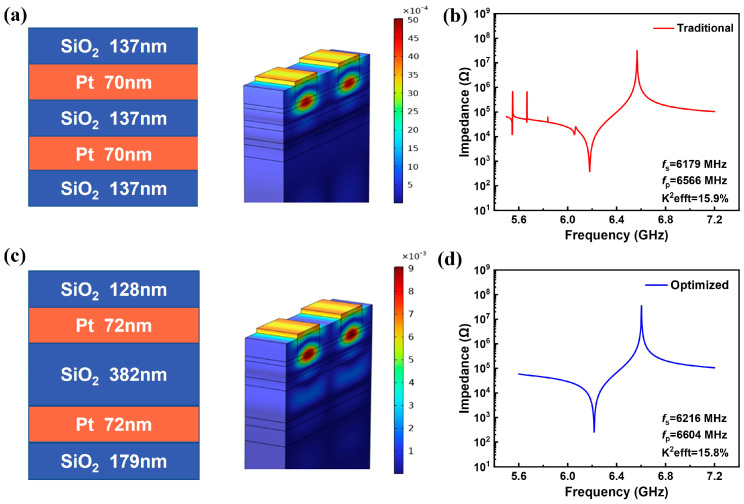
Schematic diagrams of the conventional and optimized Bragg reflector structures and impedance spectra: (**a**) conventional Bragg reflector and the surface displacement diagram of YBAR; (**b**) impedance spectrum of the YBAR with conventional Bragg reflector; (**c**) optimized Bragg reflector and the surface displacement diagram; (**d**) impedance spectrum of the optimized Bragg reflector structure.

The thickness of each layer was optimized based on the above analysis. It is worth noting that the combination of the optimal values of each layer might not achieve the best outcome, but the variation trend of these parameters can provide a guidance for continual optimization. As shown in Figure 6, considering the low acoustic impedance layers, reducing L1 and increasing L2 layers will lead to high Q factor. It was found that the thickness of the second low-acoustic-impedance layer has the most significant influence on the Q factor. The impacts of varying L2 were investigated and the variable-controlling approach was adopted by fixing L1, H1, and H2. Then, by changing the thickness coefficient L2, the optimized L2 was obtained. With the L2 fixed at the optimum value, the other thickness coefficients were further optimized. Then, we adjusted the value of L2 again, and so forth. After several round iteration, the optimized thickness of each layer was obtained. The final layers’ thicknesses and the optimized impedance curves are shown in Figure 7. Figure 7a,b show the thickness sequence of the SiO_2_/Pt stack, the vibration displacement map, and the impedance response of the YBAR with a conventional Bragg reflector design. For the optimized parameters shown in Figure 7c, a spurious-free impedance spectrum is obtained (Figure 7d). The Q values were calculated using the phase differentiation method. The resonators with the conventional λ/4 Bragg reflector has a Qs of 1502 and a Qp of 2876, and the effective electromechanical coupling coefficient is 15.9%. For the optimized structure, the Qs and Qp are 2044 and 3022 respectively, while k_eff_^2^ is 15.8%, which is comparable to that of the conventional YBAR. Comparing the impedance curves (Figure 7b,d), few spurious responses can be observed in the frequency range lower than the series resonant for the conventional YBAR. These spurious responses will result in high insertion loss when it is used to construct the filter. On the other hand, the optimized structure presents a much smoother and cleaner impedance spectral curve (Figure 7d), and a higher Q value can be obtained. To further optimize the performance, dielectric materials, such as Ta_2_O_5_ and Te_2_O_5_, can be used as the high-impedance layers to mitigate the parasitic capacitance. However, due to the relatively low ratio of acoustic impedance to Ta_2_O_5_ and Te_2_O_5_ compared to Pt or W, obtaining high reflectivity requires more reflective layers, which increases the fabrication cost.

## 6. 3D Modeling of the SM-YBAR

**Figure 8:**
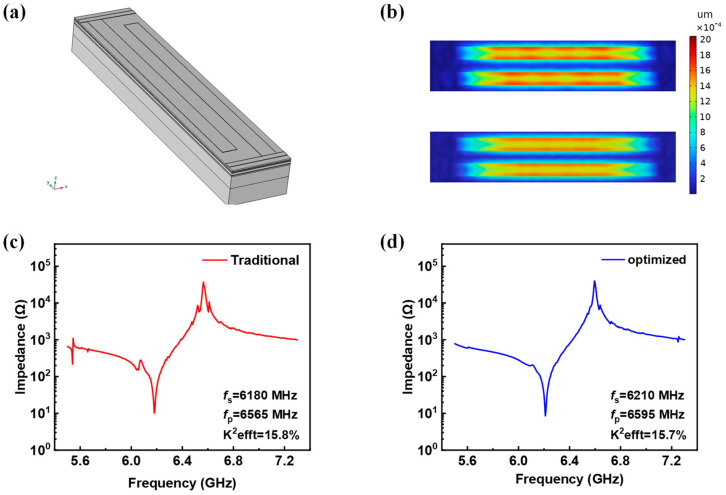
(**a**) Three-dimensional SM-YBAR structure; (**b**) top view of the surface displacements with conventional structure (top) and optimized structure (bottom); (**c**) impedance curve of conventional structure; (**d**) impedance curve of optimized structure.

To obtain more realistic results, 3D modeling was conducted. The aperture of the device was set to 40 μm, and only a pair of finger electrodes was considered. The influence of busbars was also evaluated. The busbars were set to be 1 μm wide and 50 nm thick. Periodic condition was applied along the y-direction to save the computation time, and the two surfaces normal to the *y*-axis were set to be low-reflection boundaries. In Figure 8a, the thickness scale is enlarged by a factor of five for a better view of the structure. The optimized structure and the conventional structure have the same settings except for different thicknesses of the Bragg layers. Figure 8b shows the displacement diagrams for both the conventional and optimized structures. Figure 8c,d represent impedance spectra of the YBARs with conventional and optimized structures, respectively. Consistent with the 2.5D modeling (Figure 7b,d), the optimized device presents much smoother impedance curves, fewer parasitic resonances can be observed, and intensities of spurious responses are much weaker compared to the conventional device, demonstrating the superior feature of this new design.

## 7. Conclusions

In this paper, the longitudinally excited shear acoustic wave resonators were designed using single-crystalline lithium tantalate thin film, and finite element simulations were carried out to optimize the characteristics of the acoustic resonators. Firstly, the propagation angle with the highest k_eff_^2^ of X-cut and Y-cut LiTaO_3_ YBARs was analyzed and obtained with the suspended structure. Secondly, to improve the device’s stability, a solid mounted type YBAR was designed. The optimization process for Bragg reflectors of SM-YBARs operating at over 6 GHz was carried out to improve performance of the device. A high performance SM-YBAR based on a X-cut film with the 130° propagation angle and optimized Bragg layer was obtained with high Qs and Qp values of 2044 and 3022, and k_eff_^2^ of 15.7%. Compared to the traditional Bragg reflector design withλ/4 layer thickness, Qs and Qp increased by 36% and 5%, respectively. In addition, the YBARs with optimized Bragg reflector effectively suppressed the spurious responses, and smoother impedance curve has been obtained. The proposed configuration shows great potential for next-generation communication applications.

## Figures and Tables

**Table 1:** Material parameters [14].

	Lithium Tantalate (LiTaO_3_) (Original Situation)					
Density (kg/m^3^)	7450					
Relative permittivity	$\left[\begin{array}{ccc} 40.9 & 0 & 0\\ 0 & 40.9 & 0\\ 0 & 0 & 43.3 \end{array}\right]$					
Elastic Constants (GPa)	$\left[\begin{array}{cccccc} 232.9 & 46.8 & 80.2 & -11.0 & 0 & 0\\ 46.8 & 232.9 & 80.2 & 11.0 & 0 & 0\\ 80.2 & 80.2 & 275.3 & 0 & 0 & 0\\ -11.0 & 11.0 & 0 & 93.9 & 0 & 0\\ 0 & 0 & 0 & 0 & 93.9 & -11.0\\ 0 & 0 & 0 & 0 & -11.0 & 93.0 \end{array}\right]$					
Piezoelectric Constants (C/m^2^)	$\left[\begin{array}{cccccc} 0 & 0 & 0 & 0 & 2.6 & -1.6\\ -1.6 & 1.6 & 0 & 2.6 & 0 & 0\\ 0.08 & 0.08 & 1.9 & 0 & 0 & 0 \end{array}\right]$					
	**Density (kg/m^3^)**	**Young ** **Modulus (GPa)**	**Poisson’s Ratio**	**Longitudinal Wave** **Velocity (m/s)**	**Shear** **Wave** **Velocity (m/s)**	**Acoustic** **Impedance** **(MPa/(m** **·s^−1^))**
SiO_2_	2200	70	0.17	5900	3323	12.98
Pt	21,450	168	0.38	3260	1700	69.93
Mo	10,200	312	0.31	6190	3368	63.14
Si	2329	170	0.28	8500	4700	19.20
Al	2700	70	0.33	6318	3025	17.06
Cu	8960	120	0.34	3940	2320	35.30
W	19,300	411	0.28	5200	2797	100.36

## Data Availability

The data is available on reasonable request from the corresponding author.

## References

[1] Aigner R. SAW and BAW technologies for RF filter applications: A review of the relative strengths and weaknesses. Proceedings of the 2008 IEEE Ultrasonics Symposium.

[2] Wang W., Fu Y.Q., Chen J., Xuan W., Chen J., Wang X., Mayrhofer P., Duan P., Bittner A., Schmid U. (2016). AlScN thin film based surface acoustic wave devices with enhanced microfluidic performance. J. Micromech. Microeng..

[3] Kurz N., Ding A., Urban D.F., Lu Y., Kirste L., Feil N.M., Žukauskaitė A., Ambacher O. (2019). Experimental determination of the electro-acoustic properties of thin film AlScN using surface acoustic wave resonators. J. Appl. Phys..

[4] Koulakis J., Koskela J., Yang W., Myers L., Dyer G., Garcia B. XBAR physics and next generation filter design. Proceedings of the 2021 IEEE International Ultrasonics Symposium (IUS), Xi’an.

[5] Yandrapalli S., Plessky V., Koskela J., Yantchev V., Turner P., Villanueva L.G. Analysis of XBAR resonance and higher order spurious modes. Proceedings of the 2019 IEEE International Ultrasonics Symposium (IUS).

[6] Qin Z.-H., Wu S.-M., Wang Y., Liu K.-F., Wu T., Yu S.-Y., Chen Y.-F. (2021). Solidly Mounted Longitudinally Excited Shear Wave Resonator (YBAR) Based on Lithium Niobate Thin-Film. Micromachines.

[7] Kadota M., Ishii Y., Tanaka S. (2022). 3.4 GHz strip-type thickness shear mode solidly-mounted bulk acoustic wave resonator using X-cut LiTaO_3_. Jpn. J. Appl. Phys..

[8] Guo Y., Kadota M., Tanaka S. (2023). Investigation on the temperature coefficient of frequency performance of LiNbO_3_ on quartz and glass surface acoustic wave resonators. Jpn. J. Appl. Phys..

[9] Kadota M., Yamashita F., Tanaka S. 9.5 GHz Solidly Mounted Bulk Acoustic Wave Resonator using Third Overtone of Thickness Extension Mode in LiNbO_3_. Proceedings of the 2022 IEEE International Ultrasonics Symposium (IUS).

[10] Setiawan F., Kadota M., Tanaka S. SH 1 Mode Plate Wave Resonator on LiTaO_3_ Thin Plate with Phase Velocity over 13,000 m/s. Proceedings of the 2021 IEEE International Ultrasonics Symposium (IUS), Xi’an.

[11] Kadota M., Ogami T., Yamamoto K., Tochishita H., Negoro Y. (2010). High-frequency lamb wave device composed of MEMS structure using LiNbO_3_ thin film and air gap. IEEE Trans. Ultrason. Ferroelectr. Freq. Control.

[12] Thalhammer R., Aigner R. Energy loss mechanisms in SMR-type BAW devices. Proceedings of the IEEE MTT-S International Microwave Symposium Digest.

[13] Thalhammer R., Kaitila J., Aigner R., Marksteiner S. Prediction of BAW resonator performance using experimental and numerical methods. Proceedings of the IEEE Ultrasonics Symposium.

[14] Gevorgian S.S., Tagantsev A.K., Vorobiev A.K. (2013). Tuneable Film Bulk Acoustic Wave Resonators.

[15] Jose S., Jansman A.B., Hueting R.J., Schmitz J. (2010). Optimized reflector stacks for solidly mounted bulk acoustic wave resonators. IEEE Trans. Ultrason Ferroelectr. Freq. Control.

